# Supplementary material for: The influence of habitat use on harvest vulnerability of cow elk (Cervus canadensis)
Source: PLoS One. 2020 Nov 23;15(11):e0242841. doi: 10.1371/journal.pone.0242841 (PMC7682858; doi:10.1371/journal.pone.0242841)
Supplement: S1 Table — (DOCX) [file pone.0242841.s001.docx]

|  | RuggednessScale | ElevationScale | AspectScale | DistRoadsScale | DistTreesScale | SlopeScale | DistPrivScale |
| --- | --- | --- | --- | --- | --- | --- | --- |
| RuggednessScale | 1 | -0.01483524 | 0.03614485 | 0.133671289 | -0.04975102 | 0.45849356 | 0.03275071 |
| ElevationScale | -0.01483524 | 1 | 0.029887837 | 0.122669776 | -0.52430253 | 0.07563984 | 0.31900593 |
| AspectScale | 0.03614485 | 0.02988784 | 1 | 0.001273107 | 0.01741464 | 0.05595155 | 0.02417304 |
| DistRoadsScale | 0.13367129 | 0.12266978 | 0.001273107 | 1 | 0.03121525 | 0.15939231 | 0.25305228 |
| DistTreesScale | -0.04975102 | -0.52430253 | 0.017414644 | 0.031215248 | 1 | -0.0704201 | -0.22844708 |
| SlopeScale | 0.45849356 | 0.07563984 | 0.055951553 | 0.15939231 | -0.0704201 | 1 | 0.07653346 |
| DistPrivScale | 0.03275071 | 0.31900593 | 0.024173045 | 0.25305228 | -0.22844708 | 0.07653346 | 1 |

S1 Table. Correlation values for variables used in resource selection models of cow elk in central Utah during the hunting season, calculated using ‘cor()’ function in program R.
