# Supplementary material for: The influence of habitat use on harvest vulnerability of cow elk (Cervus canadensis)
Source: PLoS One. 2020 Nov 23;15(11):e0242841. doi: 10.1371/journal.pone.0242841 (PMC7682858; doi:10.1371/journal.pone.0242841)
Supplement: Attachment — Submitted filename: PONE-D-19-22105_LFB.pdf [file pone.0242841.s003.pdf]

Manuscript

Click here to access/download;Manuscript;Harvest Vulnerability of Elk_PLOS1Submission.docx

Habitat Use and Harvest Vulnerability of Elk (*Cervus canadensis*): Do Elk Learn to Avoid Hunters as They Age?

Maksim Sergeyev[1], Brock R. McMillan[1], Kent R. Hersey[2], Randy T. Larsen[1]
[1] Department of Plant and Wildlife Sciences, Brigham Young University, Provo, UT,
[2] Utah Division of Wildlife Resources, Salt Lake City, UT, 84116, USA

Abstract:

Pressure from hunting alters the behavior and habitat selection of game species. During hunting periods, animals like deer (*Odocoileus hemionus*) and elk (*Cervus canadensis*) typically select for areas further from roads and closer to tree cover, while altering the timing of their daily activities to better avoid hunters. Our objective was to determine the habitat characteristics most influential in predicting harvest risk of elk and further, to determine if elk learned to avoid hunters with age. We captured 445 elk between January 2015 and March 2017 in the Uinta-Wasatch-Cache National Forest and surrounding area of central Utah. We determined habitat selection during the hunting season using a resource selection function (RSF). Additionally, we modeled vulnerability to harvest based on habitat use within home ranges as well as based on the location of the home range on the landscape to evaluate vulnerability on a broader scale. Elk selected for areas that reduced hunter access (rugged terrain, within tree cover, on private land). Age, elevation and distance to roads were most influential in predicting harvest risk based on use within home ranges (top model accounted for 36.2% of the weight). Elevation and distance to trees were most influential in predicting risk based on centroid of home range (top model accounted for 42.1% of the weight). Vulnerability to harvest was associated with increased proximity to roads. Additionally, survival decreased with age; we found no evidence of learned hunter-avoidance by older elk.

Introduction:

Selection of resources and habitats is a driving force influencing animal populations (Rosenzweig 1981). As such, a thorough understanding of the factors driving habitat selection is vital for proper management and conservation of a species (Lele 2009). Because resources are not uniformly available across the landscape, organisms will select the most beneficial habitats (Manly 2002). Selection occurs at multiple scales and has been categorized into specific orders of selection (Owen 1972). The broadest of these scales, first order selection, describes selection of a geographic range, while second order narrows the selection further to local sites (Johnson 1980). Third order selection describes usage patterns of local areas and finally, fourth order selection can describe selection for particular species in a diet. Selection of habitats may be influenced by quality of forage, risk of predation, energy trade-offs, or anthropogenic influences like development, outdoor recreation, and hunting (Conover 2001, Pierce et al. 2004, Nix et al. 2018)

Pressure from hunting (additional disturbance, increased risk of mortality) can influence behavior and habitat selection of game species. During hunting periods, game species often shift habitat use away from areas with optimal resource quality towards areas offering greater security (Proffitt et al. 2010). For example, black bears (*Ursus americanus*) and wolves (*Canis lupus*) shifted habitat use towards less accessible areas, further from roads (Conover 2001). White-tailed deer (*Odocoileus virginianus*) altered their habitat use and timing of daily activity to avoid hunters (Verdade 1996). Hunting led to reduced intraspecific competition, decreased mating opportunities, and increased group size in red deer (*Cervus elaphus*) and Dall sheep (*Ovis dalli*), likely due to the

removal of dominant individuals (Verdade 1996, Singer and Zeigenfuss 2002). Understanding the effects of harvest and anthropogenic activities on behavior, resource selection, and population dynamics is fundamental to conservation.

Rocky Mountain elk (*Cervus canadensis*), a big game species across the United States, similarly respond to hunting pressure suggesting that hunters may influence elk population dynamics beyond the direct effects of harvest-related mortality. During the hunting season, elk select for areas further from roads and often use private land as a means of refuge (Burcham et al. 1999, Conner et al. 2001, Viera et al. 2003, Proffitt et al. 2013). Daily movement rates increase and elk expend additional energy avoiding hunters (Johnson et al. 2004, Proffitt et al. 2009). Additionally, flight distances of elk increase during the hunt, while group size decrease, suggesting elk are acutely aware of the increased risk of mortality (Bender et al. 1999, Proffitt et al. 2009). Not only can hunting pressure influence distribution of elk, the distribution of elk on the landscape may influence susceptibility to harvest. Vulnerability of elk to harvest is likely influenced by hunter efficiency, characteristics of the home range, and detectability of the elk (McCorquodale et al. 2003). Detectability of the elk can vary with time of day and cover type and may decrease with age as older individuals become familiar with annual hunting pressure.

As elk age, they may learn the nuisances of hunter avoidance and reduce use of high-risk areas accordingly (Wright et al. 2006, Thurfjell et al. 2017). Bull elk had more pronounced responses to hunting pressure than cows and mature bulls exhibited greater flight distances than younger bulls, consistent with higher rates of harvest for mature bulls (Bender et al. 1999). Older cow elk reduced movement rates during the hunting

period and increased use of rugged terrain (Thurfjell et al. 2017). Further, the same study showed that cows over the age of 9 or 10 were less susceptible to harvest by hunters. As long-lived, gregarious animals, elk may learn to avoid hunters by altering habitat use.

The risk of harvest for a game animal is likely influenced by a multitude of factors, including selection of habitat during the hunting season. Our objectives were to determine the habitat characteristics most influential in predicting harvest risk of elk and to determine whether elk learned to avoid hunters with age. We expected risk of harvest to be correlated with hunter accessibility and that elk in rugged, less accessible areas will be at a reduced risk. Further, we predicted older elk would reduce use of high-risk areas. Identifying the factors associated with harvest risk of elk can increase knowledge of population dynamics, advance understanding of the responses of game species to hunters, and provide additional insight into age structure of the population, thereby improving management.

Methods:

Study Area

We conducted this study in the Wasatch Mountains and surrounding area of central Utah, west of Salt Lake City (Figure 1). The Wasatch range, the southwestern portion of the Rocky Mountains extending approximately 400 kilometers (Britannica 1988), is characterized by rugged terrains and steep slopes, a result of past glaciation events (Brooks 2001). The mountains are comprised primarily of dolomite and limestone (Andersen and Holmgren 1969). In addition to the rugged mountain ranges, the region

contains numerous valleys and plateaus, as well as a greater amount of domestic livestock grazing than surrounding portions of the Rocky Mountains (White et al. 2006). At a base elevation of approximately 1370 meters, Mount Nebo, at 3620 meters, is the highest point along the range, alongside other notable peaks like Mount Timpanogos and Mount Olympus (Cottam and Evans 1945, Halleran 1994). The region receives an average of 40 centimeters of annual precipitation, varying with elevation (Fuller 1973). Composition of plant communities also varies with elevation and distinct ranges have been described (Madsen and Currey 1979). Elevations below 1980 meters, the Upper Sonoran Zone, are dominated by sagebrush (*Artemisia* spp) and Mexican cliffrose (*Purshia stansburyana*), while elevations between 1981 – 2440 meters, the Transition Zone, are covered by mountain brush species like Gambel oak (*Quercus gambelii*) and curl-leaf mountain mahogany (*Cercocarpus ledifolis*; USFS 1974). The Canadian Zone, 2440 - 2900 meters, is characterized by aspen (*Populus tremuloides*) and white fir (*Abies concolor*), followed by the Hudsonian Zone, composed of subalpine fir (*Abies lasiocarpa*) and Engelmann spruce (*Picea engelmannii*). Finally, primrose (*Oenothera* spp) and alpine moss populate the Arctic-Alpine Life Zone, above 3200 meters.

Elk Capture

We captured elk via helicopter net-gunning from January 2015 through March 2017 (Webb et al. 2008). Individuals were restrained using hobble straps and fit with a blindfold, no sedation or euthanasia was involved. All capture and handling of wildlife was conducted in accordance with Brigham Young University Institutional Animal Care

and Use Committee permit #150112.We collected body measurements, blood, and fecal samples for each elk, as well as an estimate of body condition (Cook et al. 2001) and age based on dental wear. We measured loin muscle thickness and rump fat using ultrasonography. Body mass and ingesta free body fat were calculated for each individual (Cook et al. 2010). Captured individuals were then fitted with radio and GPS collars before being released. In order to balance frequency of data collected and longevity of the collars, we programmed collars to collect a GPS location every 13 hours. Mortality warnings were triggered by a lack of animal movement. When we received a mortality signal, we located the deceased animal and determined cause of death within 48 hours.

Analysis

We calculated separate home ranges for every hunting season that each elk lived through. We created 95% minimum convex polygons (MCPs) using locations during the hunting season (late August through January 31st; McCorquodale 2003, Middleton et al. 2013, Cole et al. 2015). We excluded animals with less than 50 locations during the hunting season to avoid biased estimates of home ranges (Sakai and Noon 1997, Van Dyke et al. 1998). We analyzed selection preferences during the hunting season using a resource selection function (RSF) to provide an understanding of habitat selection and distribution of individuals (Boyce et al. 2002). Based on known locations of use from collared individuals, relative probabilities of use can be estimated with an RSF (Hebblewhite et al. 2005, Lele and Keim 2006). To examine habitat use during the hunting season, we evaluated 27 candidate models of habitat selection based on all elk locations collected using an AICc model selection process for logistic regression models in program R (Akaike 1973, RCoreTeam 2013). To examine differences in selection

between day (i.e., when elk are susceptible to harvest) and night, we used interaction terms between habitat variables and a binary variable to denote the time as either day (1) or night (0).

We evaluated elk habitat use and its effect on harvest vulnerability at two scales: habitat use within home ranges (McCorquodale et al. 2003) and at a broader scale based on the overall location of the home range on the landscape using the centroid of each home range (Cole et al. 2015). We modeled risk of harvest by hunters using logistic regression with 1 corresponding to survival and 0 representing harvest (McCorquodale et al. 2003). We included variables for distance to roads, aspect, elevation, slope, terrain ruggedness, distance to tree cover, and distance to private land (McCorquodale et al. 2003, Viera et al. 2003). We evaluated vulnerability to harvest based on use within the home range by averaging data from all locations within the home range and considered each hunting season from every elk as an individual observation (Hayes et al. 2002). We excluded locations that occurred outside of hunting hours, as there was no risk of harvest mortality during these hours. We used linear mixed effects regression models to examine habitat characteristics as fixed effects while accounting for random temporal variation and dependence of the locations using animal ID as a random effect (Hebblewhite and Merrill 2008). We evaluated 20 candidate models of harvest vulnerability using an AICc selection process in program R (Akaike 1973; R Core Team 2013). To evaluate if elk learned to avoid hunters as they aged, we examined the effect of age on vulnerability. Additionally, we evaluated harvest risk based on the location of the home range on the broader landscape using the centroid of each home range (Cole et al. 2015). We obtained measurements of the aforementioned habitat characteristics for the centroid of each home

range. We evaluated the same set of 20 candidate models to compare influential habitat characteristics between the two scales. Using the top model, we developed a map of risk of hunter harvest across the study area (Kauffman et al. 2007).

Results:

Between January of 2015 and March of 2017, we captured and collared 445 elk. We restricted the analysis to locations during the hunting season and removed any elk with less than 50 locations, at which point 255 animals remained. We created separate home ranges for each hunting season during which an animal had locations, totaling 358 home ranges. We evaluated habitat selection in the context of harvest vulnerability within home ranges and on a broader scale to evaluate position of home range on the landscape. We evaluated harvest risk at two scales in order to determine vulnerability based on use within an animals home range as well as based on the overall location of the home range on the broader landscape.

Out of 27 candidate models of habitat use, the top model accounted for 83.6% of the weight compared to 16.4% for the second most supported model (Table 1). Habitat use of elk during the hunting season was influenced by aspect, elevation, ruggedness, slope, and distance to private land, trees, roads, day vs night, and an interaction between time of day and ruggedness, distance to private land, and distance to trees (Table 2). According to the interaction terms in the model, elk selected for rugged terrain, closer to private land and tree cover during the day compared to nighttime. Overall, elk selected for areas that were high in elevation and far from roads and tree cover. Steep slopes and rugged terrain were correlated with decreased use.

We determined habitat factors that had the greatest support for predicting risk of harvest and found differing results between the two scales examined. We restricted the model set to locations collected during hunting hours (30 minutes prior to sunrise – 30 minutes past sunset) as animals were at no risk of harvest outside this period. Within each animal's home range, harvest vulnerability was most influenced by distance to roads, elevation, and age of the animal (top model accounted for 36.2% of the weight, Table 3). We did not average top models because no additional statistically significant variables were present in remaining models. According to our top model, harvest risk increased with proximity to roads ($p = 0.056$, Table 4, Figure 2). Additionally, survival was lower at higher elevations (Figure 3) and for older animals (Figure 4). We examined learned hunter-avoidance by older elk using interactions terms between age and distance to roads, distance to trees, distance to private land, and elevation. We found no evidence for learned hunter-avoidance by older animals.

Based on overall location of the home range on the landscape, vulnerability to harvest was most influenced by elevation and distance to trees (top model accounted for 42.1% of the weight, Table 5). The top model included an interaction between elevation and distance to trees ($p = 0.028$, Table 6) suggesting that at higher elevations, distance to trees became more influential in predicting harvest risk. As we were unable to model age across the landscape, the top model based on home range characteristics was used to create a heatmap of harvest vulnerability across the study area (Figure 5) to illustrate high-risk areas. Our results predict high vulnerability in the northwest (Currant Creek/Wasatch front) and southwest portions (Nebo Mountains) of the study area, as well

as throughout the Uinta Mountains near the center of the study site. Additionally, we predict low vulnerability in the southeastern portion (Uinta basin).

Discussion:

Elk altered habitat selection during hunting hours, selecting for areas that limited hunter access. Habitat selection was influenced by all seven of the habitat variables measured (elevation, slope, ruggedness, aspect, distance to roads, distance to trees, and distance to private land). During hunting hours, elk selected for rugged terrain, closer to tree cover and private land. Overall, elk selected for land that was at high elevations, far from roads and further from tree cover. Additionally, we found preference for flatter, less rugged terrain. Models of habitat selection by elk typically incorporate variables describing vegetation and cover, road density, land ownership, topographical complexity, and various measures of hunter effort or access (Unsworth et al. 1998, McCorquodale et al. 2003, Cleveland et al. 2012). Similarly, our models incorporated distance to trees, distance to roads, distance to private land, slope, ruggedness, elevation, and aspect. We predicted elk would select for terrain with reduced hunter access, specifically steep, rugged terrain, within forested cover, and on private land. Based on our top model, elk altered their selection preferences during hunting hours, increasing use of areas with limited hunter access (rugged terrain, close to private land and tree cover), supporting our predictions. We also found preference for flatter, less rugged areas further from tree cover, contrary to our expectations, however during the winter elk may select flatter grasslands for forage (Proffitt et al. 2010), possibly explaining the use of flatter, open areas. During the hunting season, elk selected for rugged areas with lower road density,

closer to tree cover, consistent with other populations of elk (Hayes et al. 2002, McCorquodale et al. 2003). Additionally, we found a preference for private land, consistent with prior studies of hunted populations of elk (Burcham et al. 1999, Viera et al. 2003, Proffitt et al. 2013).

Within an animal's home range, harvest vulnerability was best predicted by distance to roads, age of the individual, and elevation. Elk had increased survival further from roads. Survival decreased with increasing elevation. This was likely due to public land generally occurring at higher elevations than private land within our study area; as hunting primarily occurred on public land, this may explain the decreased survival at higher elevations. Vulnerability of elk to harvest is often correlated with road density or proximity to roads (Millspaugh et al. 2000, Hayes et al. 2002, McCorquodale et al. 2003, Cleveland et al. 2012). Our results support the idea that harvest risk increases with proximity to roads. Additionally, survival decreased with age. We tested the idea of elk learning to avoid hunters using logistic models that included interactions terms between age and distance to roads, distance to trees, distance to private land, and elevation. However, as none of these models were among the top supported models, we found no evidence of learned behavior in regard to hunter-avoidance by older elk. In contrast, our results suggest older elk had a greater likelihood of harvest. There has been past evidence to suggest elk learn to avoid hunters with age. Mature bull elk in Michigan had greater flight distances than yearling bulls, in a population where mature bulls were harvested at five times the rate of yearling bulls (Bender et al. 1999). Older cow elk increased use of rugged terrain closer to roads (Thurfjell et al. 2017). Further, in the same study they concluded that hunter avoidance of elk improved with age through natural selection and

learning. Additional work may show patterns of hunter avoidance by elk in central Utah; however, our results provided no evidence to support this hypothesis.

Based on the centroid of the home range, risk of harvest was best predicted by distance to trees, elevation, and an interaction between the two. The interaction term was positive, suggesting that at higher elevations, survival was higher with increasing distance to trees, somewhat contradictory to our expectations. However, overlap between elk and hunters was highest in forested areas and lower in uncovered areas (Millspaugh et al. 2000), possibly explaining why we found lower harvest risk away from forest cover. Additionally, elk decreased use of forested areas during the hunting season (Cleveland et al. 2012, Thurfjell et al. 2017), consistent with our results that survival increased as distance to trees increased.

Elk altered habitat use during hunting hours, increasing use of areas with limited hunter access (rugged terrain, within tree cover and closer to private land). Additionally, elk selected for areas far from roads and high in elevation. Based on the centroid of the home range, vulnerability to harvest was influenced by elevation and distance to trees. Age, elevation and distance to roads were the best predictors of harvest risk based on habitat use within the home range. Much is known about resource selection during the hunting season, however, less research has focused on harvest vulnerability and such studies typically examine risk based on use within the home range, while our study compared vulnerability based on habitat use within home ranges and on the overall location of the home range. Further, our study benefitted from a large sample size and repetition across multiple years. However, some limitations should be taken into consideration as well. Similar studies have incorporated some measure of hunter density

or hunter effort (Millspaugh et al. 2000), which was not included in our set of variables. Other habitat variables, such as topographical complexity, that were not measured may have also been influential in predicting vulnerability to harvest. Our study supports the idea that elk select for areas with limited hunter access and highlights habitat characteristics that best predict harvest risk of elk in central Utah. These results can provide further insight into the responses of game species to hunting pressure and can be used to inform future management policies.

Acknowledgements:

This work was supported by the Rocky Mountain Elk Foundation, Sportsmen for Fish and Wildlife, Utah Division of Wildlife Resources, and Brigham Young University (Grant #R0402272). We also thankfully acknowledge Heliwild for their assistance on the wildlife captures and all of our field technicians who assisted on the project.

Literature Cited:

Akaike, H. 1973. Information theory and an extension of the maximum likelihood principle. Second International Symposium on Information Theory, Academiai Kiado: Budapest:267 - 281.

Andersen, B. A., and A. H. Holmgren. 1969. Mountain plants of northeastern Utah. Utah State University, Logan, Utah.

Bender, L. C., D. E. Beyer, and J. B. Haufler. 1999. Effects of short-duration, high-intensity hunting on elk wariness in Michigan. Wildlife Society Bulletin 27:441-445.

Boyce, M. S., P. R. Vernier, S. E. Nielsen, and F. K. A. Schmiegelow. 2002. Evaluating resource selection functions. Ecological Modelling 157:281-300.

Britannica, E. o. E. 1988. Wasatch Range. Encyclopedia Britannica.

Brooks, M. S. 2001. Wasatch and Uinta Mountain Ecoregion. Status and Trends of Land Change in the Western United States:105-112.

Burcham, M., W. D. Edge, and C. L. Marcum. 1999. Elk use of private land refuges. Wildlife Society Bulletin 27:833-839.

Cleveland, S. M., M. Hebblewhite, M. Thompson, and R. Henderson. 2012. Linking elk movement and resource selection to hunting pressure in a heterogeneous landscape. Wildlife Society Bulletin 36:658-668.

Cole, E. K., A. M. Foley, J. M. Warren, B. L. Smith, S. R. Dewey, D. G. Brimeyer, W. S. Fairbanks, H. Sawyer, and P. C. Cross. 2015. Changing migratory patterns in the Jackson elk herd. Journal of Wildlife Management 79:877-886.

Conner, M. M., G. C. White, and D. J. Freddy. 2001. Elk movement in response to early-season hunting in northwest Colorado. Journal of Wildlife Management 65:926-940.

Conover, M. R. 2001. Effect of hunting and trapping on wildlife damage. Wildlife Society Bulletin 29:521-532.

Cook, R. C., J. G. Cook, D. L. Murray, P. Zager, B. K. Johnson, and M. W. Gratson. 2001. Development of predictive models of nutritional condition for Rocky Mountain elk. Journal of Wildlife Management 65:973-987.

Cook, R. C., J. G. Cook, T. R. Stephenson, W. L. Myers, S. M. Mccorquodale, D. J. Vales, L. L. Irwin, P. B. Hall, R. D. Spencer, S. L. Murphie, K. A. Schoenecker, and P. J. Miller. 2010. Revisions of Rump Fat and Body Scoring Indices for Deer, Elk, and Moose. Journal of Wildlife Management 74:880-896.

Cottam, W. P., and F. R. Evans. 1945. A Comparative Study of the Vegetation of Grazed and Ungrazed Canyons of the Wasatch. Ecology 26:171-181.

Fuller, C. 1973. Uinta Basin. Utah History Encyclopedia.

Halleran, K. 1994. Wasatch Mountains. Utah History Encyclopedia.

Hayes, S. G., D. J. Leptich, and P. Zager. 2002. Proximate factors affecting male elk hunting mortality in northern Idaho. Journal of Wildlife Management 66:491-499.

Hebblewhite, M., and E. Merrill. 2008. Modelling wildlife-human relationships for social species with mixed-effects resource selection models. Journal of Applied Ecology 45:834-844.

Hebblewhite, M., E. H. Merrill, and T. L. McDonald. 2005. Spatial decomposition of predation risk using resource selection functions: an example in a wolf-elk predator-prey system. Oikos 111:101-111.

Johnson, B. K., A. A. Ager, J. H. Noyes, and N. Cimon. 2004. Elk and mule deer responses to variation in hunting pressure. Transactions of the Sixty-Ninth North American Wildlife and Natural Resources Conference 69:625-640.

Johnson, D. H. 1980. The Comparison of Usage and Availability Measurements for Evaluating Resource Preference. Ecology 61:65-71.

Kauffman, M. J., N. Varley, D. W. Smith, D. R. Stahler, D. R. MacNulty, and M. S. Boyce. 2007. Landscape heterogeneity shapes predation in a newly restored predator-prey system. Ecology Letters 10:690-700.

Lele, S. R. 2009. A New Method for Estimation of Resource Selection Probability Function. Journal of Wildlife Management 73:122-127.

Lele, S. R., and J. L. Keim. 2006. Weighted distributions and estimation of resource selection probability functions. Ecology 87:3021-3028.

Madsen, D. B., and D. R. Currey. 1979. Late Quaternary Glacial and Vegetation Changes, Little Cottonwood Canyon Area, Wasatch Mountains, Utah. Quaternary Research 12:254-270.

Manly, B. F. J. 2002. Resource selection by animals : statistical design and analysis for field studies. 2nd edition. Kluwer Academic Publishers, Dordrecht ; Boston.

McCorquodale, S. M. 2003. Sex-specific movements and habitat use by elk in the Cascade Range of Washington. Journal of Wildlife Management 67:729-741.

McCorquodale, S. M., R. Wiseman, and C. L. Marcum. 2003. Survival and harvest vulnerability of elk in the Cascade Range of Washington. Journal of Wildlife Management 67:248-257.

Middleton, A. D., M. J. Kauffman, D. E. McWhirter, J. G. Cook, R. C. Cook, A. A. Nelson, M. D. Jimenez, and R. W. Klaver. 2013. Animal migration amid shifting patterns of phenology and predation: lessons from a Yellowstone elk herd. Ecology 94:1245-1256.

Millspaugh, J. J., G. C. Brundige, R. A. Gitzen, and K. J. Raedeke. 2000. Elk and hunter space-use sharing in South Dakota. Journal of Wildlife Management 64:994-1003.

Nix, J. H., R. G. Howell, L. K. Hall, and B. R. McMillan. 2018. The influence of periodic increases of human activity on crepuscular and nocturnal mammals: Testing the weekend effect. Behavioural Processes 146:16-21.

Owen, M. 1972. Some Factors Affecting Food Intake and Selection in White-Fronted Geese. Journal of Animal Ecology 41:79-&.

Pierce, B. M., R. T. Bowyer, and V. C. Bleich. 2004. Habitat selection by mule deer: Forage benefits or risk of predation? Journal of Wildlife Management 68:533-541.

Proffitt, K. M., J. L. Grigg, R. A. Garrott, K. L. Hamlin, J. Cunningham, J. A. Gude, and C. Jourdonnais. 2010. Changes in elk resource selection and distributions associated with a late-season elk hunt. Journal of Wildlife Management 74:210-218.

Proffitt, K. M., J. L. Grigg, K. L. Hamlin, and R. A. Garrott. 2009. Contrasting effects of wolves and human hunters on elk behavioral responses to predation risk. Journal of Wildlife Management 73:345-356.

Proffitt, K. M., J. A. Gude, K. L. Hamlin, and M. A. Messer. 2013. Effects of hunter access and habitat security on elk habitat selection in landscapes with a public and private land matrix. Journal of Wildlife Management 77:514-524.

RCoreTeam. 2013. R: A language and environment for statistical computing. R Foundation for Statistical Computing, Vienna, Austria.

Rosenzweig, M. L. 1981. A Theory of Habitat Selection. Ecology 62:327-335.

Sakai, H. F., and B. R. Noon. 1997. Between-habitat movement of dusky-footed woodrats and vulnerability to predation. Journal of Wildlife Management 61:343-350.

Singer, F. J., and L. C. Zeigenfuss. 2002. Influence of trophy hunting and horn size on mating behavior and survivorship of mountain sheep. Journal of Mammalogy 83:682-698.

Thurfjell, H., S. Ciuti, and M. S. Boyce. 2017. Learning from the mistakes of others: How female elk (Cervus elaphus) adjust behaviour with age to avoid hunters. Plos One 12.

Unsworth, J. W., L. Kuck, E. O. Garton, and B. R. Butterfield. 1998. Elk habitat selection on the Clearwater National Forest, Idaho. Journal of Wildlife Management 62:1255-1263.

USFS. 1974. Uinta National Forest (NF), American Fork-Canyon Peak Planning Unit, Environmental Impact Statement.

Van Dyke, F. G., W. C. Klein, and S. T. Stewart. 1998. Long-term range fidelity in rocky mountain elk. Journal of Wildlife Management 62:1020-1035.

Verdade, L. M. 1996. The influence of hunting pressure on the social behavior of vertebrates. Rev Bras Biol 56:1-13.

Viera, M. E. P., M. M. Conner, G. C. White, and D. J. Freddy. 2003. Effects of Archery Hunter Numbers and Opening Dates on Elk Movement. The Journal of Wildlife Management 67:717 - 728.

Webb, S. L., J. S. Lewis, D. G. Hewitt, M. W. Hellickson, and F. C. Bryant. 2008. Assessing the helicopter and net gun as a capture technique for white-tailed deer. Journal of Wildlife Management 72:310-314.

White, D. D., M. T. Waskey, G. P. Brodehl, and P. E. Foti. 2006. A Comparitive Study of Impacts to Mountain Bike Trails in Five Common Ecological Regiosn of the Southwestern U.S. Journal of Park and Recreation Administration 24:21-41.

Wright, G. J., R. O. Peterson, D. W. Smith, and T. O. Lemke. 2006. Selection of northern Yellowstone elk by gray wolves and hunters. Journal of Wildlife Management 70:1070-1078.

Tables:

Table 1. AICc model selection results for 27 candidate models of habitat use. Top model included aspect, elevation, ruggedness, slope, distance to trees, distance to roads, and distance to trees, accounting for 83.6% of the total weight. We included Animal ID as a random effect in every model.

	d.f.	AICc	ΔAICc	Weight
Aspect + Elevation + Day*Ruggedness + Slope + DistTrees + DistRoads + Day + DistPriv + Day*DistPriv + Day*DistTrees	13	191845.2	0.00	0.836
Aspect + Elevation + Ruggedness + Slope + DistTrees + DistRoads + Day + DistPriv + Day*DistPriv + Day*DistTrees	12	191848.5	3.26	0.164
Aspect + Elevation + Ruggedness + Slope + DistTrees + DistRoads + Day + DistPriv + Day*DistPriv	11	192073.3	228.10	0.000
Aspect + Elevation + Ruggedness + Slope + DistTrees + DistRoads + DistPriv+ Day + Day*DistTrees	11	192214.2	368.97	0.000
Null	1	193528.7	1683.48	0.000

Table 2. Output from top model (based on AICc) of habitat selection of elk during the hunting season.

	Estimate	Std. Error	p – Value
Intercept	-0.0045	0.0073	0.533
Aspect	0.0634	0.0054	< 0.001
Elevation	0.0394	0.0066	< 0.001
Day	0.0133	0.0109	0.221
Ruggedness	-0.0448	0.0077	< 0.001
Slope	-0.1368	0.0062	< 0.001
DistTrees	0.0992	0.0084	< 0.001
DistRoads	0.0881	0.0059	< 0.001
DistPriv	0.0078	0.0080	0.331
Day*Ruggedness	0.0251	0.0109	0.022
Day*DistPriv	-0.2163	0.0113	< 0.001
Day*DistTrees	-0.1663	0.0111	< 0.001

Table 3. AICc model selection results for 20 candidate models of survival based on habitat use. We included Animal ID as a random effect in every model. Models with greater than five percent of the cumulative weight are listed below. Top model included age, distance to roads, and elevation, accounting for 36.2% of the total weight.

	d.f.	AICc	ΔAICc	Weight
Age + DistRoads + Elevation	5	283.4	0.00	0.362
DistRoads + Elevation + Age + DistPriv	6	284.7	1.37	0.195
Age + Elevation + DistRoads + Ruggedness	6	285.5	2.07	0.129
Elevation + DistRoads + Aspect + Slope + Age + DistPriv	8	286.1	2.45	0.096
Age + Elevation	4	286.3	3.21	0.084
Null	1	295.1	11.71	0.001

Table 4. Output from top model (based on AICc) of harvest vulnerability of elk based on habitat use.

	Estimate	Std. Error	p – Value
Intercept	1.937	0.172	< 0.001
Age	-0.114	0.151	0.451
DistRoads	0.465	0.243	0.056
Elevation	-0.305	0.179	0.089

Table 5. AICc model selection results for 20 candidate models of survival based on overall location of the home range on the landscape. We included Animal ID as a random effect in every model. Models with greater than two percent of the cumulative weight are listed below. Top model included elevation, distance to trees, and an interaction term, accounting for 42.1% of the total weight.

	d.f.	AICc	ΔAICc	Weight
Elevation + DistTrees + Elevation*DistTrees	5	239.0	0.00	0.421
Elevation + Ruggedness	4	242.1	3.15	0.087
Age + Elevation	4	242.3	3.33	0.080
Age*DistRoads + Age + DistRoads	5	242.5	3.52	0.072
Age*Elevation + Age + Elevation	5	242.8	3.87	0.061
Age + DistRoads + Elevation	5	243.7	4.71	0.040
Age + DistTrees + DistPriv	5	243.8	4.79	0.038
Age*DistTrees + Age + DistTrees	5	243.8	4.81	0.038
DistPriv + DistTrees	4	244.3	5.33	0.029
Age*DistPriv + Age + DistPriv	5	244.4	5.38	0.029
Age + Elevation + DistPriv	5	244.4	5.38	0.029
Slope + Aspect + Ruggedness + DistTrees	6	244.6	5.61	0.025
Elevation + DistTrees + Ruggedness + DistRoads	6	244.9	5.95	0.021
Null	1	295.1	56.16	0.000

Table 6. Output from top model (based on AICc) of harvest vulnerability of elk based on based on overall location of the home range on the landscape.

	Estimate	Std. Error	p – Value
Intercept	10.784	1.447	< 0.001
Elevation	0.430	0.811	0.596
DistTrees	0.507	0.804	0.528
Elevation*DistTrees	1.228	0.560	0.028

Figure
Click here to access/download;Figure;Harvest Vulnerability of Elk_Fig1.docx

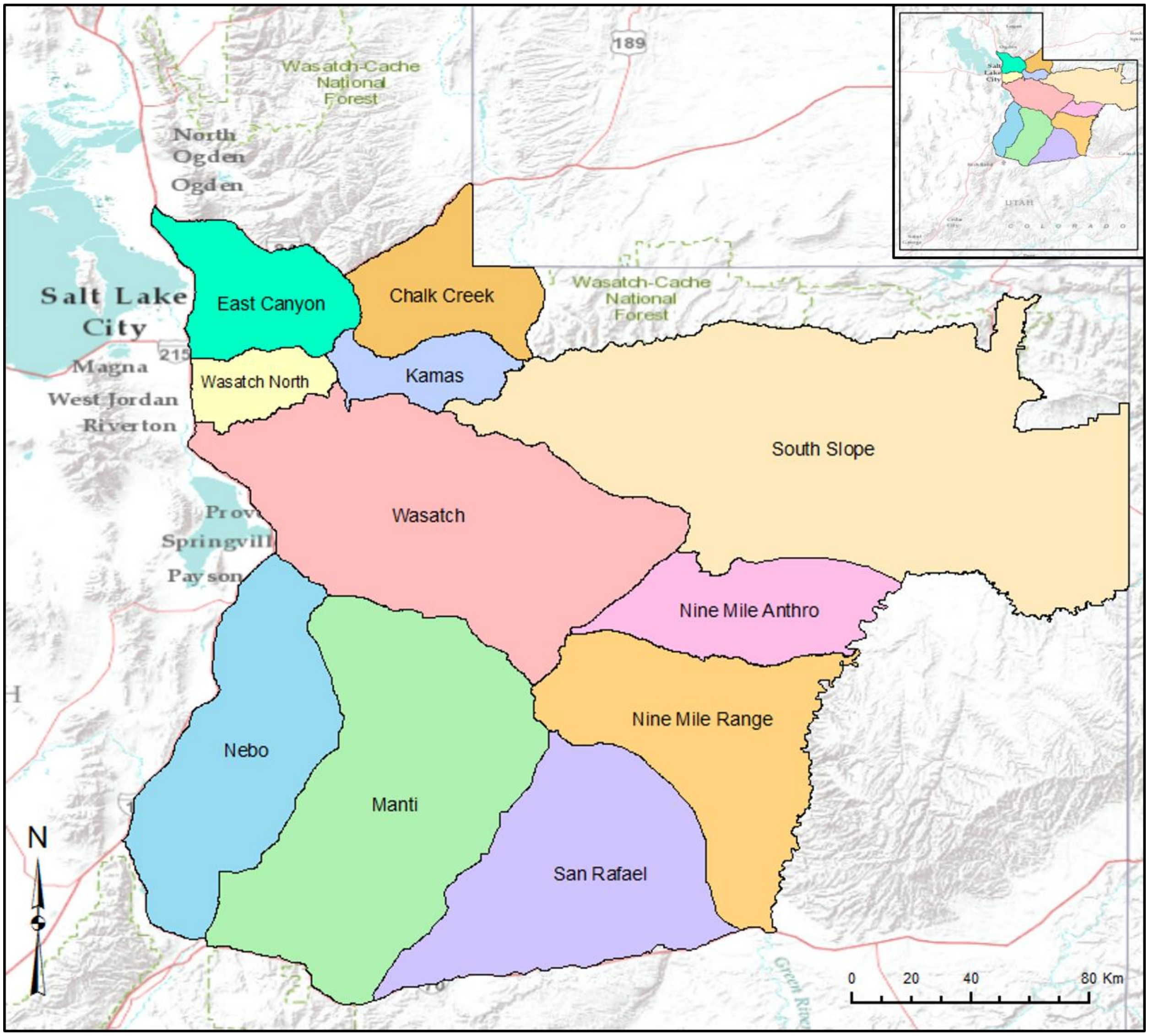

Figure 1. Our study area was the Wasatch and surrounding management units of central Utah. Colored polygons denote the separate management units.

Figure

Click here to access/download;Figure;Harvest Vulnerability of Elk_Fig2.docx

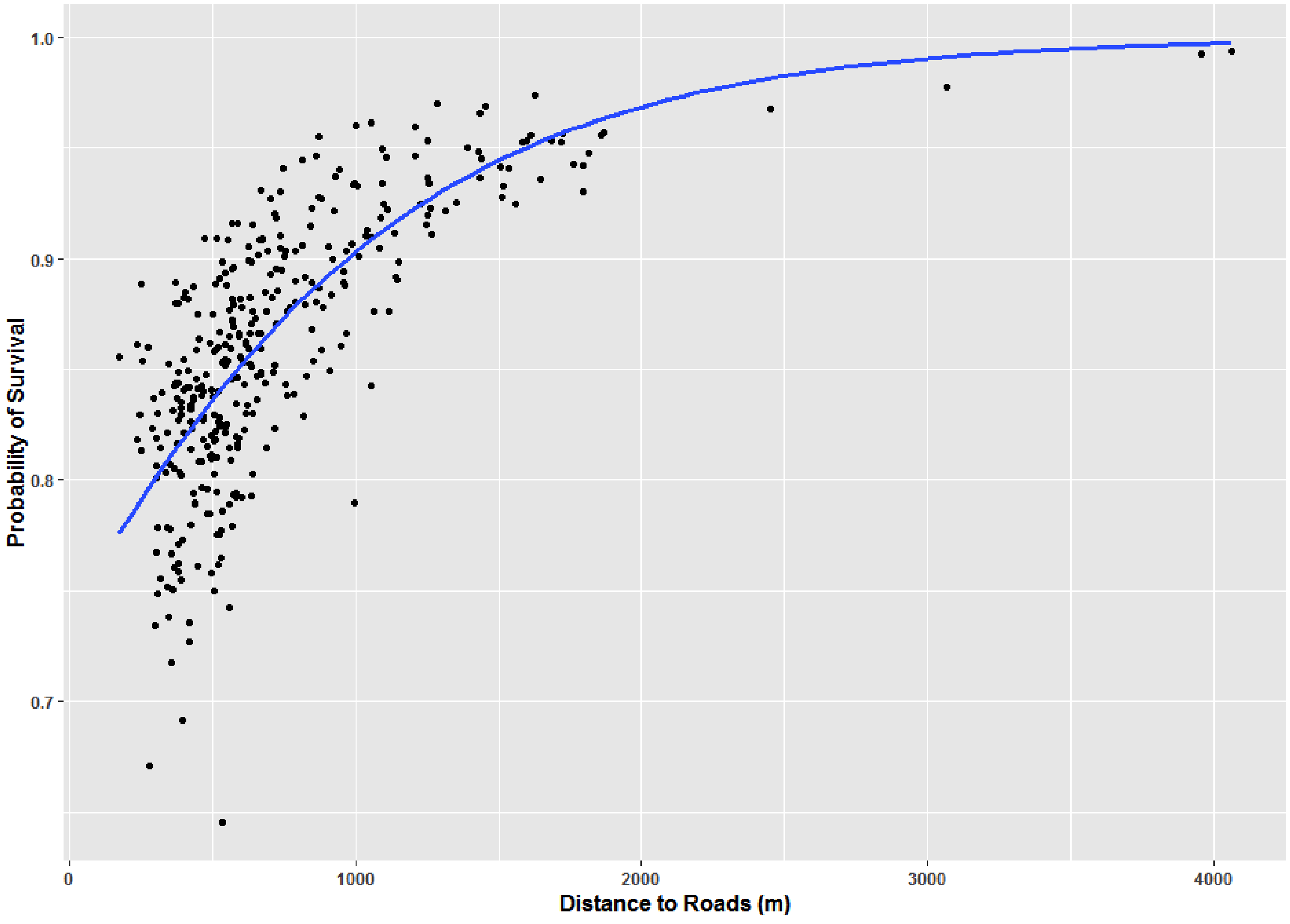

Figure 2. Predictive model of harvest vulnerability based on distance to roads, according to the top model from AICc selection. Top model included age, distance to roads, and elevation.

Figure

Click here to access/download;Figure;Harvest Vulnerability of Elk_Fig3.docx

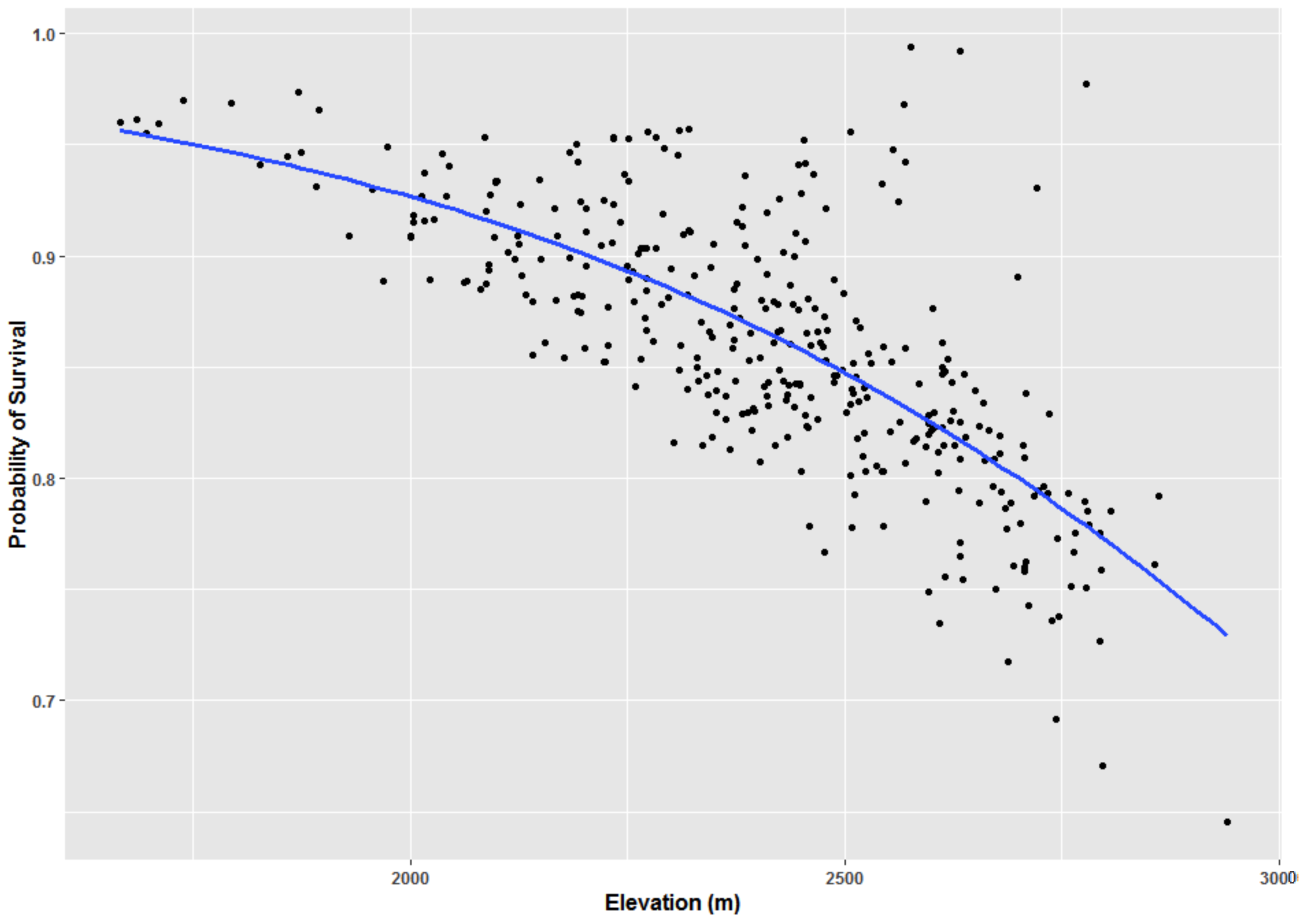

Figure 3. Predictive model of harvest vulnerability based on elevation, according to the top model from AICc selection. Top model included age, distance to roads, and elevation.

Figure Click here to access/download;Figure;Harvest Vulnerability of Elk_Fig4.docx

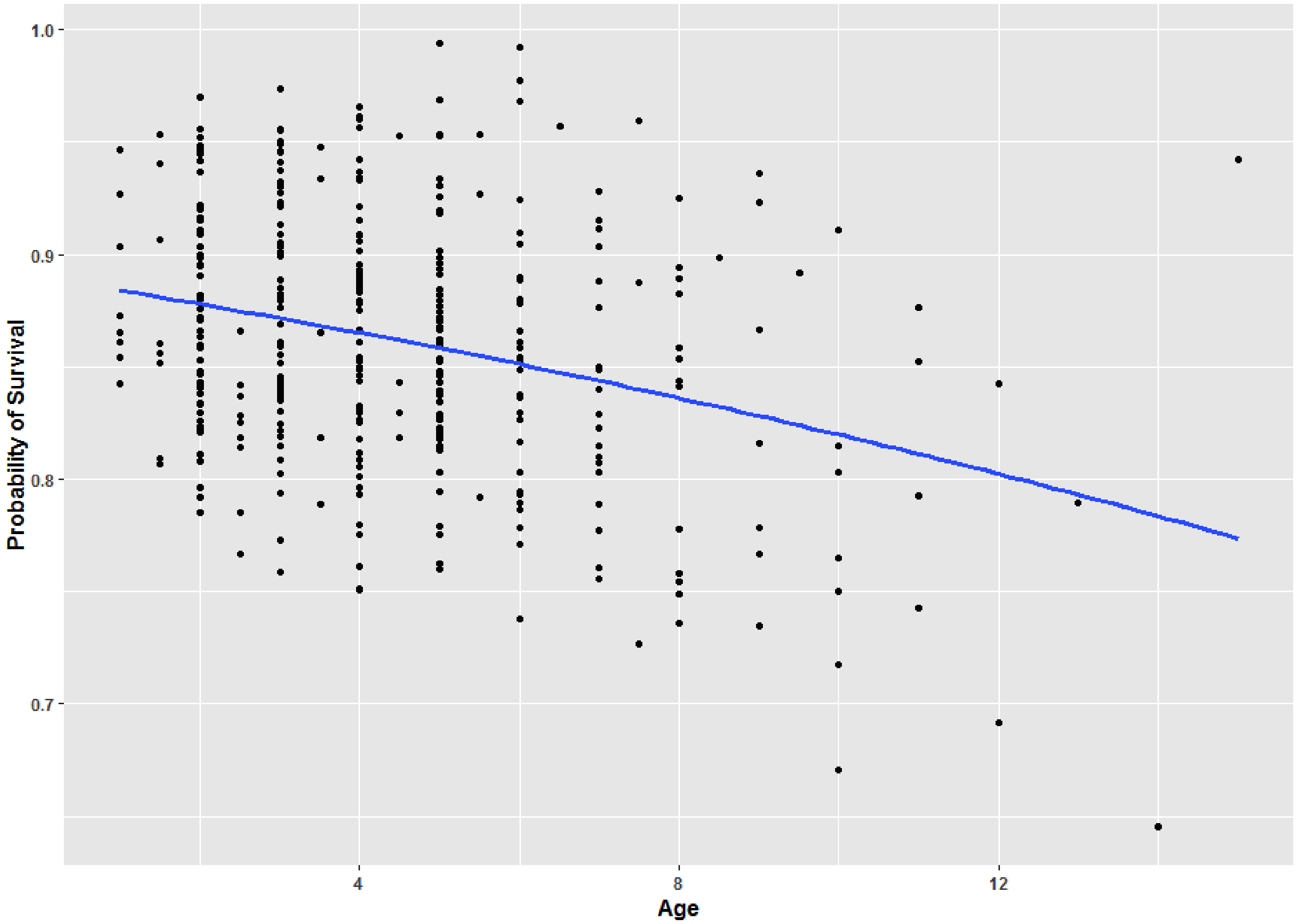

Figure 4. Predictive model of harvest vulnerability based on age, according to the top model from AICc selection. Top model included age, distance to roads, and elevation.

Figure

Click here to access/download;Figure;Harvest Vulnerability of Elk_Fig5.docx

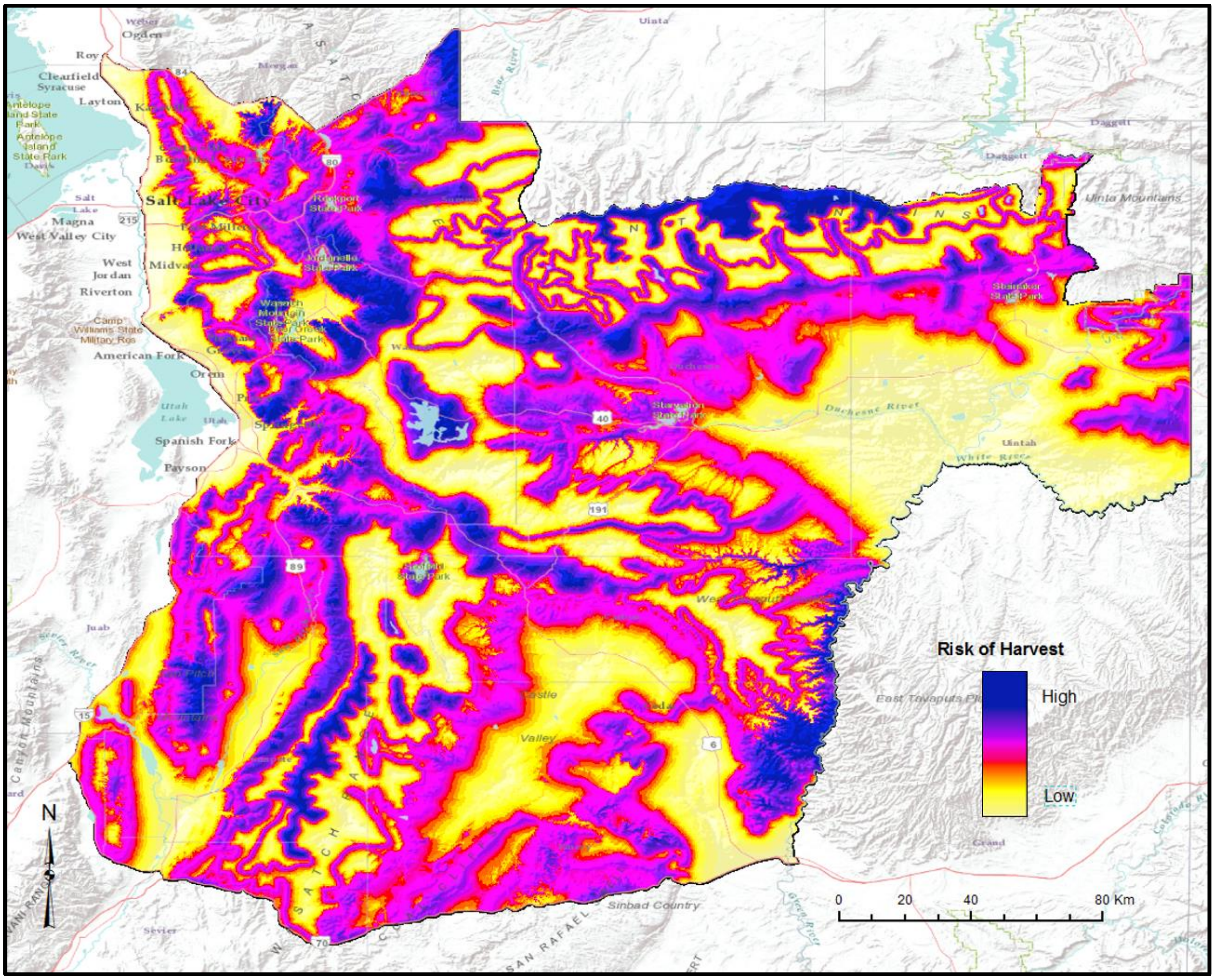

Figure 5. Heat map of elk harvest vulnerability based on the location of the home range on the landscape, modeled as a function of elevation, distance to trees, and an interaction between the two.
