# Supplementary material for: The influence of habitat use on harvest vulnerability of cow elk (Cervus canadensis)
Source: PLoS One. 2020 Nov 23;15(11):e0242841. doi: 10.1371/journal.pone.0242841 (PMC7682858; doi:10.1371/journal.pone.0242841)
Supplement: Attachment — Submitted filename: Response to Reviewers 05.03.2020.docx [file pone.0242841.s004.docx]

Maksim Sergeyev

Department of Plant and Wildlife Sciences

Brigham Young University

Provo, Utah 84602

ecomaksimsergeyev@gmail.com

847-404-2103

March 1, 2020

Dear Editors and Reviewers,

The following manuscript is being resubmitted following comments made by the editor and peer reviewers. We are very grateful for the additional revisions. We believe the comments provided addressed some weaknesses in the document and resulted in a more thorough and complete manuscript. Specifically, we have added some additional information regarding hunting practices and objectives in the Western United States, clarified some details in our methods and analysis, and performed model validation for the models of habitat use. In addition, we have included a link to an online repository with the data used for the analysis. The edits that were made are further clarified below.

Thank you to the editor and reviewers for your time and consideration. We look forward to your response.

Sincerely,

Maksim Sergeyev

1. Please ensure that your manuscript meets PLOS ONE's style requirements, including those for file naming. The PLOS ONE style templates can be found at <http://www.plosone.org/attachments/PLOSOne_formatting_sample_main_body.pdf> and <http://www.plosone.org/attachments/PLOSOne_formatting_sample_title_authors_affiliations.pdf>

*We corrected issues in formatting, including adding page and line numbers and correcting file names of the figures.*

2. In your Methods section, please provide additional information on the blood collection and ensure you have included details on (1) locations of where the blood was collected, and (2) the volume of collected blood from each animal.

*We added some additional text to the ‘Elk Capture’ subsection of the Methods section clarifying the amount of blood that was collected and the location on the animal from where it was collected.*

3. We note that Figures 1 and 5 in your submission contain map/satelliteimages which may be copyrighted.

All PLOS content is published under the Creative Commons Attribution License (CC BY 4.0), which means that the manuscript, images, and Supporting Information files will be freely available online, and any third party is permitted to access, download, copy, distribute, and use these materials in any way, even commercially, with proper attribution. For these reasons, we cannot publish previously copyrighted maps or satellite images created using proprietary data, such as Google software (Google Maps, Street View, and Earth). For more information, see our copyright guidelines: <http://journals.plos.org/plosone/s/licenses-and-copyright>.

We require you to either (a) present written permission from the copyright holder to publish these figures specifically under the CC BY 4.0 license, or (b) remove the figures from your submission:

a. You may seek permission from the original copyright holder of Figures 1 and 5 to publish the content specifically under the CC BY 4.0 license.

We recommend that you contact the original copyright holder with the Content Permission Form (<http://journals.plos.org/plosone/s/file?id=7c09/content-permission-form.pdf>) and the following text:

“I request permission for the open-access journal PLOS ONE to publish XXX under the Creative Commons Attribution License (CCAL) CC BY 4.0 (<http://creativecommons.org/licenses/by/4.0/>). Please be aware that this license allows unrestricted use and distribution, even commercially, by third parties. Please reply and provide explicit written permission to publish XXX under a CC BY license and complete the attached form.”

Please upload the completed Content Permission Form or other proof of granted permissions as an "Other" file with your submission.

In the figure caption of the copyrighted figure, please include the following text: “Reprinted from [ref] under a CC BY license, with permission from [name of publisher], original copyright [original copyright year].”

b. If you are unable to obtain permission from the original copyright holder to publish these figures under the CC BY 4.0 license or if the copyright holder’s requirements are incompatible with the CC BY 4.0 license, please either i) remove the figure or ii) supply a replacement figure that complies with the CC BY 4.0 license. Please check copyright information on all replacement figures and update the figure caption with source information. If applicable, please specify in the figure caption text when a figure is similar but not identical to the original image and is therefore for illustrative purposes only.

The following resources for replacing copyrighted map figures may be helpful:

USGS National Map Viewer (public domain): <http://viewer.nationalmap.gov/viewer/>

The Gateway to Astronaut Photography of Earth (public domain): <http://eol.jsc.nasa.gov/sseop/clickmap/>

Maps at the CIA (public domain): <https://www.cia.gov/library/publications/the-world-factbook/index.html> and <https://www.cia.gov/library/publications/cia-maps-publications/index.html>

NASA Earth Observatory (public domain): [http://earthobservatory.nasa.gov/](http://earthobservatory.nasa.gov/%20)

Landsat: <http://landsat.visibleearth.nasa.gov/>

USGS EROS (Earth Resources Observatory and Science (EROS) Center) (public domain): [http://eros.usgs.gov/#](http://eros.usgs.gov/)

Natural Earth (public domain): <http://www.naturalearthdata.com/>

*We added the copyright watermarks from ESRI in the bottom corner of Figures 1 and 5 that should address any issues over copyrights.*

4. Thank you for including your ethics statement:

"Capturing and handling of animals was conducted in accordance with Brigham Young University Institutional Animal Care and Use Committee permit #150112. Animals were not anesthetized or euthanized."

a. Please amend your current ethics statement to confirm that your named ethics committee specifically approved this study.
b. Once you have amended this/these statement(s) in the Methods section of the manuscript, please add the same text to the “Ethics Statement” field of the submission form (via “Edit Submission”).

For additional information about PLOS ONE submissions requirements for animal ethics, please refer to <http://journals.plos.org/plosone/s/submission-guidelines#loc-animal-research>.

*We edited the text in the methods section to clarify that our procedures were all approved by the BYU IACUC.*

5. We note that you have indicated that data from this study are available upon request. PLOS only allows data to be available upon request if there are legal or ethical restrictions on sharing data publicly. For more information on unacceptable data access restrictions, please see <http://journals.plos.org/plosone/s/data-availability#loc-unacceptable-data-access-restrictions>.

In your revised cover letter, please address the following prompts:

a) If there are ethical or legal restrictions on sharing a de-identified data set, please explain them in detail (e.g., data contain potentially sensitive information, data are owned by a third-party organization, etc.) and who has imposed them (e.g., an ethics committee). Please also provide contact information for a data access committee, ethics committee, or other institutional body to which data requests may be sent.

b) If there are no restrictions, please upload the minimal anonymized data set necessary to replicate your study findings as either Supporting Information files or to a stable, public repository and provide us with the relevant URLs, DOIs, or accession numbers. For a list of acceptable repositories, please see <http://journals.plos.org/plosone/s/data-availability#loc-recommended-repositories>.

We will update your Data Availability statement on your behalf to reflect the information you provide.

*We have uploaded the data used for modeling habitat use and harvest vulnerability to our laboratory website and have provided a link below. We are unable to share any GPS coordinates but have provided the habitat variables for each location.*

6. Please include a separate caption for each figure in your manuscript.

*We added any missing captions for figures included in the manuscript and made sure that file names were in accordance with the submission guidelines.*

[Note: HTML markup is below. Please do not edit.]

Reviewers' comments:

Reviewer's Responses to Questions

**Comments to the Author**

1. Is the manuscript technically sound, and do the data support the conclusions?

The manuscript must describe a technically sound piece of scientific research with data that supports the conclusions. Experiments must have been conducted rigorously, with appropriate controls, replication, and sample sizes. The conclusions must be drawn appropriately based on the data presented.

Reviewer #1: Partly

Reviewer #2: Partly

2. Has the statistical analysis been performed appropriately and rigorously?

Reviewer #1: No

Reviewer #2: Yes

3. Have the authors made all data underlying the findings in their manuscript fully available?

The [PLOS Data policy](http://www.plosone.org/static/policies.action#sharing) requires authors to make all data underlying the findings described in their manuscript fully available without restriction, with rare exception (please refer to the Data Availability Statement in the manuscript PDF file). The data should be provided as part of the manuscript or its supporting information, or deposited to a public repository. For example, in addition to summary statistics, the data points behind means, medians and variance measures should be available. If there are restrictions on publicly sharing data—e.g. participant privacy or use of data from a third party—those must be specified.

Reviewer #1: No

Reviewer #2: Yes

4. Is the manuscript presented in an intelligible fashion and written in standard English?

PLOS ONE does not copyedit accepted manuscripts, so the language in submitted articles must be clear, correct, and unambiguous. Any typographical or grammatical errors should be corrected at revision, so please note any specific errors here.

Reviewer #1: Yes

Reviewer #2: Yes

5. Review Comments to the Author

Please use the space provided to explain your answers to the questions above. You may also include additional comments for the author, including concerns about dual publication, research ethics, or publication ethics. (Please upload your review as an attachment if it exceeds 20,000 characters)

**Reviewer #1**: General comments
Deer populations are traditionally managed by means of hunting. Deer may respond to hunting by using refuges, avoiding human activity centers, modifying movement, activity and habitat selection. Accordingly, knowledge of the effects of hunting on deer behavior is necessary to enhance the effectiveness of deer management. In the present manuscript you determine the habitat characteristics most influential in predicting harvest risk of elk and if elk learned to avoid hunters with age. Although I do not call the relevance of these data into question, I have several points that need to be clarified or refined but see my general and specific comments.

**Methods**
PLOS is an international journal and not all readers are familiar with elk hunting in the USA. In this regard, you should include additional information in Methods (see points below):
How are elk hunted in the Wasatch Mountains and surroundings? Are there different types of hunting (e.g. hunting with beaters, hunting from a hide…)? Please specify

Could you please provide further information about how elk hunting is organized and if there is a shooting plan (number of males, females and fawns)? If there is a shooting plan you should add it in Methods.
All this information will be useful for the interpretation of your results and for the discussion.

*We added two additional paragraphs to the methods section to address this comment and the preceding one. These sections are under included under an additional subheading entitled “Elk Hunting in the Western United States”, intended to provide some additional background information on hunting practices and objectives in the Western US.*

Could you please provide more information about what is exactly meant by private land and why you think that this variable should be included to model elk habitat use and risk of harvest? I am especially wondering why private land should be less accessible to hunters given that some private landowners could be hunters too or allow hunters to hunt on their ground according to my understanding. Clarify please.

*We added additional text to the ‘Analysis’ subsection of the Methods section. When describing the variables included in the study, we clarified the inclusion of distance to private land by explaining use of private land by game species as a means of refuge from hunting pressure.*

**Study design and statistical analyses:**
You wrote “We analyzed selection preferences during the hunting season using a resource selection function”. For me it is not clear what you aim for by doing so. The chosen approach does not enable to draw any conclusions regarding the hunting effect as you cannot be sure that elk alter habitat selection during hunting hours because of hunting or because of other confounding factors (see below).
If all elk included in the present study are exposed to hunting pressure, it precludes a comparison of hunted with non-hunted individuals in order to detect hunting effects.

*Unfortunately, we were unable to compare hunted individuals with a non-hunted population as all elk within the area are susceptible to hunter harvest. While other factors may certainly confound habitat use of elk, we analyzed differences between daytime hours, when risk of harvest was present, compared to nighttime, when no risk of harvest was present. In this manner, we can examine differences in habitat use when risk of harvest is present versus absent. Other factors are likely still influential, however, predation is generally low and little competition exists with other ungulates and therefore, we felt that this comparison should provide an idea of how habitat use changes in response to the presence of hunters.*

Comparing habitat selection during the hunting season to the period before or after is not ideal as seasonal trends in selection and changes in behavior due to hunting can be confounded.
To disentangle other effects (e.g. human disturbances, predators, competition with domestic animals…) from hunting effects, you need to choose a different approach. You could e.g. follow the method described in Behr et al. (2017) and build two separate habitat selection models using two nested data sets: the full data set which includes all location data over the entire year (all data model) and the reduced data set in which you exclude the hunting period from the data and interpolate elk habitat use during the missing hunting period (no-hunting model). As stated by Behr et al. (2017) this method could fail to disentangle a seasonal from a hunting effect if the seasonal driver of habitat selection would perfectly coincide with the hunting season. Hence you need to check if there is a correspondence between the natural history of elk and the timing of the hunting season. If there is no correspondence, you are on the save side.

*Habitat selection by elk is likely greatly confounded by seasonal effects in Utah. The heavy snows will typically push elk to lower elevations during the colder periods, while availability of forage and extreme heat likely influence habitat use during the summer. For this reason, we restricted our analysis to the hunting season to minimize these seasonal differences. Rather than compare a period before and after the season, we chose to compare hours of harvest risk to hours absent of hunting pressure. In this way, seasonal differences would be minimized while providing opportunity to draw conclusions about differences in habitat use in response to hunters.*

I recommend to take gender into account in the analyses as vulnerability to harvest based on habitat use could differ between males and females.

*After restricting our analysis to the hunting season and to individuals with over 50 locations, only females remained in our data set. We clarified this detail in the results section.*

You should provide more information on how elk were aged and on how many age categories were considered in the analysis.

*We provided some additional details in the methods section regarding our methods for aging elk (based on dental wear).*

I recommend to screen for collinearity between predictors prior to the analysis. Check please (see also my specific comments).
I recommend to perform some validations e.g. Spearman rank correlation from the k-fold cross-validation.

*Predictor variables were screened for any issues of collinearity, no variables had high correlation values. We performed a Spearman rank correlation to validate our models of habitat use, the resulting p-value is included in the Results section.*

**Formatting of the manuscript:**
You should ease the work of the reviewers and include line and page numbers.

*We added page and line numbers to the document to make the review process easier.*

**Data availability:**

*We uploaded the data files used for the analysis to an online repository on GitHub. The link to that data is below:*

<https://github.com/MaksimSergeyev/HarvestVulnerabilityElk>

**Specific comments**
Major comments
**Introduction**
Page 4: Other aspects such as vigilance, site familiarity could influence the risk of harvest as well. I suggest adding them here. Risk of harvest does not only depend on hunter accessibility other factors could be important as well. E.g. once a deer is spotted/discovered the probability to be harvested could depend on sex, antler size, body condition. It depends furthermore on the type of hunting and on the hunting plan (see above). Clarify please.

*We provided some additional details on hunting plans, means of take, and hunting objectives in an additional subsection in the Methods that describes some of the issues described regarding which animals are harvested and how. In regard to the condition and size related attributes, we analyzed these factors in another analysis based on these same elk. In that paper, we examine cause-specific mortality and how condition and size related attributes differ between elk harvested by hunters and those predated by mountain lions. This paper is currently in the revision process at Journal of Wildlife Management and will help provide additional information on the effects of hunting and differences between predation by large carnivores versus human hunters.*

Page 4: Besides hunting other factors could influence elk habitat selection including disturbances, interspecific competition (e.g. competition with domestic animals) and predators. In these regards, how can you be sure that the observed difference in habitat selection between night and day is solely the effect of hunting and not due to other confounding factors? Clarify please.

*While numerous factors will likely always confound habitat use by elk, we feel comfortable that our analysis draws valid conclusions about how behavior differed between times of harvest risk compared to when harvest was not present. We restricted our analysis to the hunting season to minimize seasonal effects from changing temperature, weather, and vegetation availability. We then compared habitat use between locations collected when hunting was present versus absent by examining interaction terms in our model between Day (1 during hunting hours and 0 outside of hunting hours) with landscape variables such as ruggedness, distance to trees, and distance to private land. In this way, we examined whether habitat use changes in regard to these variables when hunters are present versus absent.*

Could you, please provide more information about the domestic livestock (page 5: Methods/Study area:)? Which species, herding practices (are they grazing in fenced pastures), is livestock staying out at night, etc.? Could they potentially compete with elk? Clarify please and discuss.

*We removed the portion that mentioned livestock from the methods as this was irrelevant to the rest of the paper. There is likely very little competition for resources between domestic livestock and elk as elk typically live in montane landscapes away from urban centers while agriculture typically occurs at lower elevations in flatter, open areas.*

Are predators such as cougar and wolves present in your study area (these species are manly nocturnal) and could they have a confounding effect with hunting? Clarify please and discuss.

*Wolves and bears are absent from the area, however, cougars are present. Predation of elk by cougars is generally minimal and typically cougars target young or old individuals in poorer condition. We analyzed mortalities by cougars and compared age, size, and condition to those predated by hunters in a paper on cause-specific mortality in this same population. Results showed very little overlap between elk taken by hunters and those predated by cougars. This paper should be available soon, it is currently in review at Journal of Wildlife Management.*

Even if you could exclude livestock and predators there could be other confounding factors you do not know about and hence it could be worthwhile to use an approach that enable to disentangle hunting from other effects (see my suggestion above).

*The effects of cougar predation and livestock in the area are likely very minimal. It is very difficult to completely remove other factors that influence habitat use by elk, however, we believe our approach highlights differences that can confidently be attributed to the effects of hunting.*

**Methods**
Page 6: “…and age based on dental wear”. Knowing that age estimation by means of dental wear are not very accurate, I was wondering, which age categories were considered in the present study? Clarify please.

*We evaluated age as a continuous variable where each individual was aged by multiple observers and estimated to a single year. We added additional information to the Methods section describing the aging process and the accuracy of such techniques.*

Page 6: “minimum convex polygon” Why did you chose MCP knowing that these can include large proportion of unsuitable habitat especially in a human dominated landscape? I would have expected Kernel home ranges. Please justify why you used MCP in the present study.

*We chose to use MCPs as a means of creating a boundary within which to evaluate habitat selection, rather than a means of estimating a home range. We felt comfortable using 95% MCPs because the area occupied by elk was away from human landscapes or other uninhabitable areas and, as such, our MCP estimates did not include any areas that were unsuitable habitat for elk.*

**Analysis**
This is section is not well presented and needs some reordering. I recommend adding subtitles before each section. According to the information provided in the manuscript, I see three topics/sections:
1) Habitat selection to examine habitat use during the hunting season by taking into account differences in selection between day and night. Here it would be worthwhile to choose an approach that enables to disentangle hunting from other confounding effects (see my suggestion above).
2) Evaluation of vulnerability to harvest within the home range including learned hunter-avoidance by older elk.
3) Evaluation of vulnerability to harvest based on the overall location of home range on the landscape including learned hunter-avoidance by older elk.

*We separated our analysis section into 3 subsections as recommended. We agree with the comment that this helps with clarity and readability.*

Page 6: “Based on known locations of use…Lele and Keim, 2006). Please provide further details regarding the sampling design used in the present study fort both within home range and overall location of home range on the landscape. For example, how did you asses availability (number of random locations), etc…See for example Hebblewhite et al. (2005).

*We used an equal number of random locations to used locations for each elk. We confirmed that this was adequate by computing the true means of each variable within the area used and confirming that the random locations encompass these true means within a 95% confidence interval.*

Page 6 to page 7: “To examine differences in selection between day …and night…” how did you define day and night in your study? Did you consider that days length changes over the course of the study? Clarify please and provide this information in the manuscript.

*We defined ‘day’ as hours when hunting was present: 30 minutes prior to sunrise until 30 minutes after sunset, as mandated by hunting regulations in the state of Utah, and considered ‘night’ as any time outside of these hours. We did adjust this window as sunrise and sunset changed over the course of the study period (hunting season).*

Page 7: “We modeled risk of harvest by hunters using logistic regression with 1 corresponding to survival and 0 to harvest…”
In case if elks were harvested during different type of hunting in the present study, I think that it would be wise to consider different categories in the analyses. Clarify please.

*We analyzed both hunting seasons together because we felt that the overall presence of hunters on the landscape would have a greater effect on behavior than specific hunting types. Literature on these species suggests that elk are acutely aware of the increased activity and greater risk of mortality and, as such, we believe that they would likely respond similarly based on the increased anthropogenic disturbance and risk of mortality during both seasons. We added text to the methods section to describe grouping the hunting seasons into one study period.*

I suspect that the harvest risk of a deer varies over the course of the day (highest probability just before dusk and after dawn) and study period (more deer are shot at the beginning of the hunting period then towards the end) and depends as well on the characteristics (sex, age, condition) of each individual. You did not consider these aspects in the present manuscript.

*In regard to characteristics such as condition and age, this was beyond the scope of this analysis, however, we analyzed these factors in a separate analysis that is currently under journal review. In that paper, we detail how age, size, and condition differ between elk harvested by hunters, those predated by cougars, and the overall population. In regard to sex, once the data was restricted to the hunting season and animals with greater than 50 locations, only female elk remained in the study pool and as such, we were unable to draw conclusions about differences between males and females. In regard to time of day, there is no way to measure when animals are shot or when the majority of hunters are on the landscape, however, hunting in Utah is a very popular recreational sport and hunters are likely present on the landscape throughout the majority of the available hunting hours.*

In these regards, could you please provide data about the number of deer (males and females) that are present in your study area, the proportion of collard individuals (males and females) and the proportion of individuals, present in the study area, which are shot each year.

*Data on number of animals harvested, population objectives by management unit, hunter success rates, and similar metrics are available on the Utah Division of Wildlife Resources website.*

Among the harvested individuals could you please provide information about the sex and age.

*We address these topics in another paper on cause-specific mortality, in which we analyze how age, size, and body condition influence risk of harvest within this same population of elk. In this analysis, we focused more on habitat use and how that correlated with risk of harvest.*

Could you please provide further information about the distribution of the number of deer shot over the daily cycle and study period in a graph.

*In a separate paper on survival and cause-specific mortality, we have figures that detail survival throughout the year and include trend lines factoring in hunter mortality and excluding hunter mortality to highlight differences in number and timing of hunter related mortalities and how survival changes as a result. This paper is currently under review and should be available soon.*

If you have access to the locations of the deer that are shot each year you could use these data to validate your heat map of elk harvest vulnerability.

*Unfortunately this data is unavailable. We only have access to the number of permits given out within an area and the total number harvested within that unit (on the UDWR website as mentioned above), however, specific information such as location or timing of the kill was unavailable.*

Page 7: “We included variables for distance to roads, aspect, elevation, slope, terrain ruggedness, distance to tree cover and distance to private land.”

Could you please provide further details regarding the source of the variables used in the present study and their resolution, ideally in a table?

Could you please explain how these variables were sampled in the GIS?

*We provided additional details in the ‘Analysis’ section of the Methods regarding where these data were obtained and how these variables were calculated in GIS.*

Please provide the size of the radius around each location for the habitat use within home range and the size of the radius around the centroid of each home range for the habitat use at a broader scale based on the overall location of the home range on the landscape.

*We used the exact locations, without radii. The elevation raster used to compute many of the other rasters was 30 x 30 meter resolution.*

I do not understand why you used the centroid and did not calculate e.g. the average elevation within each MCP? Clarify please.

*We used the centroid of the MCP to examine how the overall location of the home range on the landscape affected harvest risk. By evaluating characteristics of the center of the range, we could examine risk at a broader scale and compare this to use within the home range to examine whether the overall position on the landscape predisposed certain individuals to a greater risk than others regardless of use within the range. We used the averages from the season to evaluate vulnerability based on 3^rd^ order use.*

Which road categories were considered in the analysis (main roads, forest roads, hiking trails…)? Clarify please.

*We combined forest roads and main roads into a single road layer and calculated distance based on this combined layer. During hunting seasons, forest roads are used by hunters and, as such, likely influence habitat use by elk. We clarified this detail in the methods section.*

How did you calculate aspect? Did you use it as a categorical value in the analysis? Clarify please.

*We calculated aspect from the digital elevation model raster. Aspect was computed as a value from 0 – 360 and included as a continuous variable. We added this detail in the methods section.*

Page 7: “We evaluated vulnerability to harvest based on use within the home range by averaging data from all locations within the home range and considered each hunting season from every elk as an individual observation (Hayes et al. 2002).” Not too clear, see also my comment regarding the sampling design used in the present study. Clarify please.

*Locations from each elk for each specific hunting season were all averaged to create a data file that contained one row for each season that an individual lived through, with a status as harvested or not and average values for each of the variables measured. From this, we modeled vulnerability.*

**Results**
Page 8: How many females and males did you collars?

*We updated the results to include number of animals caught by sex.*

Could you please summarize the number of females and males collared per age category in a table (elk ID, sex, age, date of capture, date of loss, reason for loss, survey period (number of days), number of fixes)?

*In this analysis we only included animals lost to hunter harvest. Some of the information requested above is beyond the scope of this paper, however, we have more detailed information on survival and cause-specific mortality available in another paper addressing those topics that is currently in review. That paper details the losses from all sources of mortality, condition and size of animals lost, and includes survival figures that show timing of loss throughout the year. The supplemental data for that paper includes the number of days monitored and the reason for loss.*

Do MCP sizes differ between males and females? If yes, you should take this into consideration in the analysis.

*Once the data was cleaned and we restricted the analysis to the hunting season and only animals with > 50 locations, only female elk remained. As such, no MCPs were constructed for males.*

Why did you pool the sexes in the analyses?
I suspect that harvest vulnerability based on habitat varies between sexes (males are more vulnerable than females because of their antlers). Hence, I recommend to take gender into account in the analyses. Do you know if females had calves or not? If yes, you could consider this in the analyses as well. Clarify please.

*Please see prior comments regarding sex. We only included females and clarified this detail at the beginning of the results section.*

Page 8: “Steep slopes and rugged terrain were correlated with decreased use.“ Why did you not screen for collinearity between predictors prior to the analysis? Clarify please.

*Predictor variables were screened for collinearity prior to analysis. This sentence was meant to say that those types of terrain were associated with decreased use; we clarified the wording to reflect this.*

Page 9: “We restricted the model set to locations collected during hunting hours (30 minutes prior to sunrise – 30 minutes past sunset) as animals were at no risk of harvest outside this period.” This is not a result and should be moved to the appropriate section in Methods.
Why did you consider a duration of 30 minutes any justification? Clarify please.

*We used these restrictions because these were the legal hunting hours in the state of Utah. Risk of harvest was only present during these hours and therefore we divided our locations as occurring within these hours or outside of these hours.*

Page 9: “The top model included an interaction between elevation and distance to trees (p = 0.028, Table 6) suggesting that at higher elevations, distance to trees became more influential in predicting harvest risk.” Could you please provide further information on how (direction) harvest risk varied with elevation and distance to trees? Please add this information in the manuscript.

*We clarified the direction of the estimate for this term in the Results section and discussed this estimate in the Discussion section.*

Page 9: “As we were unable to model age across the landscape, the top model based on home range characteristics was used to create a heatmap of harvest vulnerability across the study area (Figure 5) to illustrate high-risk areas.” In contrary to the habitat selection within home ranges you did not provide any result here regarding learned hunter-avoidance by older elk at the broader scale, is there a reason? Clarify please.

*We added text to the Results section stating that we found no evidence of learned hunter avoidance based on centroids of home ranges.*

**Discussion**
Page 11: “This was likely due to public land generally occurring at higher elevations than private land within our study area; as hunting primarily occurred on public land, this may explain the decreased survival at higher elevations.” In this regard you should have screened for collinearity between predictors prior to the analysis.

*Predictor variables were screened for collinearity prior to analysis, no problematic correlations were observed.*

Page 11: “Mature bull elk in Michigan had greater flight distances than yearling bulls, in a population where mature bulls were harvested at five times the rate of yearling bulls (Bender et al. 1999).” This is not really connected to the topic of the present manuscript. This is a behavioral difference and has nothing to do with habitat selection and hence should be removed from the discussion

*We included this reference as an example of elk altering their behavior with age to reduce risk of harvest, to provide additional context that, while we did not observe learned hunter avoidance, this behavior has been observed in other areas.*

.
Page 12: “Additional work may show patterns of hunter avoidance by elk in central Utah…” Not clear what you mean here. Clarify or remove please.

*We clarified this text in the Discussion section. This was meant to say that we found no evidence of such behavior, however, future work may demonstrate that elk in central Utah alter their behavior with age in order to reduce susceptibility to harvest.*

Page 12: “Based on the centroid of the home range, risk of harvest was best predicted by distance to trees, elevation, and an interaction between the two. The interaction term was positive, suggesting that at higher elevations, survival was higher with increasing distance to trees…” This belongs to results and hence should be moved to this section. Adjust please.

*We relocated this text to the methods section.*

Page 13: “These results can provide further insight into the responses of game species to hunting pressure and can be used to inform future management policies.” This statement is too general. Here you should make some clear and concrete recommendations on how your results can be used to inform management policies. According to your findings what adjustments would be needed in the current management policies? Adjust please.

*We added some text to clarify that by better understanding vulnerability across the landscape, allocation of hunting permits can be adjusted to more accurately meet population objectives.*

Minor comments
**Introduction**
Page 2: “Selection of habitats may be influenced by …” you could add inter- and intraspecific competition as well here.

*We added ‘competition’ into the list of potential factors influencing habitat use.*

**Methods**
Page 6: “late August through January 31st” Could you please provide the exact period?

*We clarified the dates of the hunting season. The opening date varied annually but always occurred at the end of August. The closing date was always Jan. 31^st^. We clarified this detail in the text.*

**Results**
Page 8: “We evaluated habitat selection in the context of harvest vulnerability within home ranges and on a broader scale to evaluate position of home range on the landscape. We evaluated harvest risk at two scales in order to determine vulnerability based on use within an animals home range as well as based on the overall location of the home range on the broader landscape.” Both sentences have the same meaning. Moreover, this section belongs to Methods and hence should be deleted as these aspects were already mentioned in the corresponding section.

*We adjusted this accordingly and relocated the text to the methods section and reduced redundancy between sentences.*

Page 8: “Overall, elk selected…” Not clear what you mean by overall and to which results (e.g. Table) you are referring to here? Clarify please.

*We added a citation to a specific table to clarify which specific results we are referencing here.*

Page 9: I suggest changing “Additionally, survival was lower at higher elevations (Figure 3) and for older animals (Figure 4).” to “Additionally, harvest risk was higher at higher elevations (Figure 3) and for older animals (Figure 4).” Because you specifically looked at harvest risk and not overall survival which could include other mortality risk as well. This comment is valid for the whole manuscript. Please check throughout the manuscript and adjust where necessary.

*We chose this wording because, while we were interested in harvest risk, we modeled survival with data only including harvest mortalities. As such, the results from the model were describing survival based on these variables, the inverse of this would be harvest vulnerability and therefore we could interpret vulnerability by the opposite direction of the estimates generated.*

Table 1, legend: “AICc model selection results for 27 candidate models of habitat use.” Only five models are provided here. What is meant by “Day”? Clarify and adjust please.

*We clarified the ‘Day’ variable in the legend and specified which models were included in the table.*

Table 2, legend: What is meant by Day? Clarify please.

*We clarified the ‘Day’ variable in the legend.*

Table 3, legend: “AICc model selection results for 20 candidate models of survival based on habitat use.” Only six models are provided here. Clarify and adjust please.

*We clarified the legend to state why only 6 of the 20 models were included (based on AICc weight).*

Table 5, legend: “AICc model selection results for 20 candidate models of survival based on overall location of the home range on the landscape. Only 14 models are provided here. What is meant by “Day”? Clarify and adjust please.

*We clarified which models were included in the table (based on AICc weight). The Day variable was not included in these models and is not mentioned in the table.*

Figure 1: What is the additional information provided by the inset? Instead I suggest showing the location of the study area in the USA. Adjust please.

*We included the inset map of the state of Utah to better place our study within the context of the state. While the location of Utah within the United States can be found, the location of our study within the state may be more difficult to place without the map and for this reason we included the inset map showing our study area within the state boundary.*

Reference cited in this review not already cited in the present manuscript
Gehr, B., E. J. Hofer, M. Pewsner, A. Ryser, E. Vimercati, K. Vogt, and L. F. Keller. 2017. Hunting-mediated predator facilitation and superadditive mortality in a European ungulate. Ecology and Evolution 8:109–119.

**Reviewer #2**: Very interesting paper, well written and the statistical analyses seems up to date. One thing that comes to mind when looking at your study is that other studies seem to account for the sex of the animal. I am not familiar with the exact policies in Utah, but my experience from elk hunting suggests that hunters have different attitudes towards hunting females and males, often looking for trophies in males, and meat in females (and spikes). Trophies come at a relative high age, also proportion of tags may differ compared to what is available for each sex. I am not sure it is possible, but it would be nice to see if it is possible to distinguish differences if the analyses were re run, once with males and once with females. A study from Alberta by Ciuti et al. (2012), together with the paper by Thurjell et. al that you already cite suggests that, at least in Alberta, the high hunting pressure on males leads more to a selection of traits than a learning experience. There is also the issue of how gregarious animals are during hunting season, it is hard to imagine a learning process without members of a group being shot. As males are less gregarious than females, we would expect a different process there (even though effects has been found in bulls in Michigan as you state).
There are also other studies that suggest lower mortality with age (Wright 2006), thus it would be interesting if you made some attempt to dive in to the differences compared to those studies. (By more detailed analysis as suggested, or at least by a discussion on the subject).

*We clarified details in the Results section to state that this analysis only included female elk. We added the Wright 2006 study mentioned in the comment in the Discussion section.*

As for the hunting season, from late August to Jan 31st is quite a long time, with quite different weather conditions for the elk (And different weapons and tactics used by hunters, as Utah has an archery season and a Muzzel loader season). Yet I see no attempt to account for that, there are several ways to do this. If you want to account for seasonality and still have the effect of hunting (as they will be mixed up), one way of doing this is to add a factor weekend or weekdays, as we could expect hunting pressure is generally higher on Saturdays and Sundays.

*We did not separate hunting season by weapon type as we believed that the overall presence of hunters on the landscape and the heightened risk of mortality would similarly influence elk behavior. In addition, as hunting in Utah is a very popular recreational sport, there is likely hunting pressure present throughout the week as well as weekends. As such, we believed that this ever-present hunting pressure would be influencing habitat use of elk regardless of season or weekend versus weekday. We did, however, separate the locations collected as occurring within or outside of hunting hours to compare periods of harvest risk to those without risk of harvest. We also added the Cuiti 2012 study to the Discussion section.*

As for minor details such as language and grammar, as a non native English speaker, that is not my forte, and I tried to focus on the structure here.

Ciuti S, Muhly TB, Paton DG, McDevitt AD, Musiani M, Boyce MS. Human selection of elk behavioural traits in a landscape of fear. Proc R Soc B-Biol Sci. 2012;279(1746):4407–16.
Wright GJ, Peterson RO, Smith DW, Lemke TO. Selection of northern Yellowstone elk by gray wolves and hunters. J Wildlife Manage. 2006;70(4):1070–8.

*Thank you for all of the comments provided. We feel that incorporating these edits has helped with clarity and readability while providing additional relevant details on our methodology, study area, and results.*
