# Supplementary material for: The influence of habitat use on harvest vulnerability of cow elk (Cervus canadensis)
Source: PLoS One. 2020 Nov 23;15(11):e0242841. doi: 10.1371/journal.pone.0242841 (PMC7682858; doi:10.1371/journal.pone.0242841)
Supplement: Attachment — Submitted filename: Harvest Vulnerability of Elk_Resubmission R2R.docx [file pone.0242841.s005.docx]

**Reviewer #1:**

General comments

Although the manuscript has been improved. There are still several points that need further attention. Especially the manuscript is technical not sound as there are several problems in your sampling design and statistical analyses that preclude making sound conclusions at the current stage. But see my general and specific comments below.

In this analysis you apparently only included animals lost to hunter harvest (as you have written in your answers to reviewers).Could you please justify (rational behind) why you only used individuals lost to hunter harvest? Beside that this is not clearly stated in the material and method, I think that you should include all elk cows followed by means of GPS telemetry over the course of this survey in the analyses including those that were not shot over all the hunting seasons, as done by McCorquodale et al. (2003). Check their paper to see exactly how they proceed. Exclusion of animals that survived throughout all hunting seasons might bias our analysis toward elk using less secure environments. Clarify please and adjust if necessary.

*I apologize for any confusion, we did indeed include surviving elk. We only removed any elk that were lost to sources of mortality other than harvest so that we can more confidently apply our conclusions to hunter harvest as opposed to overall mortality. As such, our analysis included elk lost to hunters and any that survived the monitoring period.*

In this regard you should summarize the number of cows and bulls collared per age category in a table (elk ID, sex, age, date of capture, date of loss, reason for loss (hunting, predation,…), survey period (number of hunting seasons, number of days, number of fixes). Moreover, you should add in the results how many elk died and how many deaths were hunting related (like McCorquodale et al. 2003).

*We have included a data file summarizing the monitoring period, age, mortality status and cause of death for all monitored females as supplementary material. As data on males was very limited and males were excluded from our analysis, we excluded these animals from the table as well. We have included the number of hunter related mortalities in the results section. More detailed information on survival and cause-specific mortality is included in another paper from this study that will be published shortly. In that paper, we detail all of the sources of mortality, examine size and condition related variables in the context of mortality, and determine differences in selection between human hunters and mountain lions.*

You mentioned that habitat selection by elk is likely greatly confounded by seasonal effects in Utah. The heavy snows will typically push elk to lower elevations during the colder periods, while availability of forage and extreme heat likely influence habitat use during the summer. For this reason, you restricted our analysis to the hunting season to minimize these seasonal differences. Given that the hunting season stretches from the end of August and is concluded on January 31st, it encompasses the transition from autumn to winter that include significant changes e.g. heavy snows will typically push elk to lower elevations during the colder periods, with snow elks can easier be detected by hunters thanks to the tracks they leave in the snow. Hence as the hunting season stretches over quite a long period, with quite different weather conditions for the elk and different weapons and tactics used by hunters resulting in changes in the hunting success you need to account for that as already recommended by the other reviewer in the analysis.

*We have added language to the methods section and discussion section addressing potential issues with confounding effects of changing weather patterns or variation in hunting pressure throughout the season or by weapon type. We acknowledged that the observed differences may be due, in part, to other factors. However, each elk (whether it survived or was harvested) was treated the same in our analysis and thus any biases associated with our analysis spanning the summer-winter period are consistent across elk. We also noted that a large sample size and repetition between years may add credence to our conclusions and indeed identify differences in habitat selection as a response to increased risk of mortality from hunters.*

You excluded animals with less than 50 locations during the hunting season to avoid biased estimates of home ranges. Given that your interest in estimating home ranges was principally to define sampling frames for quantifying characteristics of areas used by elk (rather than a means of estimating a home range as stated in your response to reviewer’s comments), I do not see why you should discard data from elk that were killed before a large sample of relocations were obtained. Exclusion of such animals might bias your analysis toward elk using higher-security environments. Clarify and discuss please.

*While we were indeed using the locations as a boundary in which to evaluate habitat selection, there were instances in which poor collar success rate resulted in very few locations during this period. As such, we felt that any conclusions about habitat selection based on so few of locations may be biased or inaccurate. Further, with so few locations we may not have had an accurate idea of where the animal was for much of the hunting season. We therefore established a threshold of 50 locations based on criteria set forth in past studies. In this way, we would have sufficient locations to confidently estimate the animal’s position on the landscape and conduct resource selection analyses on these locations.*

Variables were screened for collinearity in program R (line 207). Which analysis did use to test for multicollinearity between predictors and what was the threshold? Please provide the results of this analysis as a supplementary material.

*We tested for collinearity using the ‘cor()’ function in R, which calculates a value from -1 to 1 based on the extent of correlation between the two variables. Any values more extreme than +/- 0.6 were considered problematic. This output has been included as supplementary material.*

Could you please provide more details on how you proceeded with the validation (lines 214-215)? How many folds did consider? Clarify please.
Furthermore, I could not find any values of the Spearman’s rank correlation in Table 1 (lines 260-261). What is the value of the Spearman’s rank correlation? Clarify please.
Why do you just do a validation for the “habitat use during hunting season” but not for the remaining two analyses “harvest vulnerability based on 3rd order habitat use” and “harvest risk based on location of home range”. Clarify please.

*We added the number of folds used for cross validation to the methods section and included our value for the rank correlation in the results section. We were unable to apply this same methodology to validate the survival models in the second and third sections due to differences in response variables. We were unable to find suitable methods to validate these mixed-effect survival models, however, we included the AICc values and model weights in the tables and used these metrics as a way of determining strength of the top model compared to the competing models.*

As your study focus solely on elk cows you need to adjust the title and abstract and the main text accordingly. In the discussion you should focus mainly on findings from other studies related to elk cows, especially if differences between bulls and cows are expected.

*We have adjusted the title and body of the manuscript to reflect that the analysis only pertained to cow elk and have included some additional studies that better relate to our topic.*

You indicated that wolves and bears are absent from the area, however, cougars are present and stated that predation of elk by cougars is generally minimal and typically cougars target young or old individuals in poorer condition. Even though predation of elk by cougars is minimal, even low carnivore densities affect prey behaviour (Kuijper et al. 2016). Besides, I question that cougars target old individuals in poorer condition. I would have rather expected to see such a pattern with wolves. Cougars are stalk and ambush predators and hence have a higher probability to catch less alert prey and exert selection pressure rather on the behavior of their prey (less alert prey have a higher chance to be predated) while wolves, because of the way they hunt, exert selection pressure rather on the condition of their prey. If you were not able to take such confounding effects into account in the analyses you should at least discuss this shortcoming in the discussion of the manuscript.

*We have added some additional text to the discussions section addressing the shortcomings in regard to seasonal and annual variation, variation between hunting types, and addressed that a multitude of factors likely impact habitat selection of elk, including presence of predators. In regard to prey selection by cougars, we examined this idea in a paper on survival and cause-specific mortality and observed selection for individuals at the extremes of the age distribution and in leaner condition than average. However, in total, predation was consistently low across the study, we totaled 16 mortalities related to cougars between 2015 and 2018.*

You wrote that you examined whether habitat use changes in regard to these variables when hunters are present versus absent. Yes, but what if in your study area period of times when hunters are present versus absent overlap with other factors such as disturbances and predators. In this case you cannot distinguish hunting from these confounding effects. You should at least discuss this shortcoming in the discussion of the manuscript.

*We have added language at the end of the discussion section to address the shortcomings mentioned above, such as variation in weather, weapon type, annual variation between seasons, and the possibility of other factors influencing habitat type such as presence of predators.*

No track changes are visible in the document “Revised Manuscript with Track Changes”. Please make sure that you upload the right files to ease the work of the reviewers.

*We apologize for that omission, we were careful to retain any changes made for this revision and have uploaded a separate file with changes noted.*


Specific comments

Lines 121-123: Could you please specify roughly the period in months of the archery only, muzzleloader only, and any weapon hunting seasons?

*We’ve added language to the methods section clarifying these differences.*

Lines 173-175: Did you calculate an MCP for each hunting season that an elk lived/was followed by means of GPS telemetry? Clarify please

*A separate MCP was created for each season that an elk lived through, if an animal survived multiple seasons then multiple MCPs were created. We clarified this detail in the methods section.*

Lines 190-192: This sentence can be deleted as this information appears earlier.

*We moved this sentence to an earlier part of the methods where it is more relevant and reduced redundancy.*

Lines 198-203: In the last sentence you repeat what you have already written in the first sentence. Shorten please

*We have moved the citations from the last sentence to the first sentence and remove the last sentence altogether.*

Lines 223-226: You repeat what you have written in the previous subchapter. Hence this part can be shortened considerably.

*We have edited this sentence to clarify the difference in this section compared to the prior section and reduced the length of this sentence, as well.*

Lines 306-312: Here you repeat the results provided at line 300-302. The whole section starting from line 300 to 312 should be substantially simplified and streamlined. Please rewrite it.

*We shortened these few sentences and removed some redundancy and unnecessary information.*

Line 322: “Survival decreased with increasing elevation.” Could also be related to snow cover!? Clarify please and discuss if deemed important.

*This was likely due to a greater amount of public land occurring at higher elevations, where hunting is more prevalent. We added a sentence to acknowledge a potential impact from snowfall.*

Lines 336-339: This sentence is not really connected to the topic of the present manuscript. This is a behavioral difference and has nothing to do with habitat selection. Moreover, you restricted your analyses to elk cows. Hence this sentence should be removed from the discussion.

*We removed the sentence regarding bull elk from the discussion.*

Lines 343-345: This sentence can be removed as it says nothing substantial

*This sentence was removed.*

Lines 372-374: It is not clear what you mean exactly here. Could you please further develop this idea. In other words, how would you proceed to allocate hunting permits?

*We expanded upon the implications of the study by adding some additional text to that sentence and adding an additional one at the end.*

Reference cited in my review

Kuijper D P J,Sahlen E,Elmhagen B,Chamaille-Jammes S,Sand H,Lone K, et al. Pawswithoutclaws? Ecological effects of largecarnivores in anthropogenic landscapes. Proceedings of the Royal Society B: Biological Sciences 2016; 283: 20161625. <https://doi.org/10.1098/rspb.2016.1625> PMID: 27798302

**Reviewer #2:**

Now that it is clear that this is not an overall elk-study, I think the title and onwards should be clearly stated as female Elk instead of just elk. This follows through the manuscript.

*We altered the title and language throughout the abstract and manuscript to better reflect that this analysis only pertained to female elk.*

Line 167-172 is a justification for methods used, this is more fitting in the introduction, it also suggests that there is literature on game species about awareness of risk etc… this needs to be referenced directly after the statement.

*We relocated these sentences to the final paragraph of the introduction, in the section describing the study and the objectives.*

177-180, do not mention concerns and how you feel in the materials and methods section, describe which method you choose and why, that’s it.

*We altered the language in this sentence to more objectively explain what we did and the justification for it.*

234 Was the modelled random effect regarding slope or intercept or both, and why did you select that way? Compare to Thurfjell 2017 where the formulation of the random effect was used to test if there was a learning effect.

*The random effect of animal ID was used as a random intercept. We incorporated this variable as a random intercept to account for any dependence between repeated observations of the same individual. We carefully examined the methodology used in Thurfjell 2017 and believe that some key differences exist between these studies. Specifically, we had a larger sample size but with shorter monitoring duration, making comparisons of an individual’s behavior as it ages more difficult, as many elk were only captured in year 2 or 3, and many from year 1 did not survive the entirety of the study or experienced collar failure. The response variables between the two studies differed as well. Rather than modeling use of rugged terrain or forest cover as response variables, we incorporated these as predictor variables in modeling differences in habitat use between periods of risk and without risk, and incorporated these land-use characteristics in survival models. In addition, while the study area in Thurfjell 2017 included areas with differing hunting regimes, the elk in our study area were all subjected to the same hunting regime (i.e. no areas existed where hunting was absent). The overall questions of whether habitat use changes in regard to harvest risk and if this behavior and the likelihood of survival differ with age are similar between our study and the Thurfjell study. However, we attempted to answer these questions in a slightly different method by using interaction terms to compare landscape variables between day and night to evaluate habitat selection and interaction terms between landscape variables and age to determine if older animals were altering their behavior as a result of learned hunter avoidance.*

255 How often were locations taken? What was the success rate?

*We mention in the methods section that locations were collected every 13 hours. Success rate varied greatly between individuals, with some collecting largely every point expected and others having gaps of weeks or months or failing at some point during monitoring.*


In the discussion, I lack some hypothesis as to why female elk did not show learning, as opposed to precious studies, also why do you think they are more vulnerable at older ages? Think a bit about these results, and try and formulate something that can be useful for further studies.

*We’ve added several sentences to the discussion to address this issue. Thank YOU!*
