# Supplementary material for: The influence of habitat use on harvest vulnerability of cow elk (Cervus canadensis)
Source: PLoS One. 2020 Nov 23;15(11):e0242841. doi: 10.1371/journal.pone.0242841 (PMC7682858; doi:10.1371/journal.pone.0242841)
Supplement: Attachment — Submitted filename: Harvest Vulnerability of Elk_Resubmission Resp2Rev.docx [file pone.0242841.s006.docx]

**Reviewer #1:**

General comments

Line 22: change "Elk" to "female Elk"

*We have corrected the wording from ‘elk’ to ‘female elk.’*

Line 30: change "Elk" to "female Elk"

*We have corrected the wording from ‘elk’ to ‘female elk.’*

Lines 362-363: You did not study Elk activity. Please adjust the sentence accordingly

*We adjusted the text and corrected the statement from ‘behavior and activity’ to ‘habitat use,’ which is more accurate to our study.*

Lines 378-379: I do not see the rational given that harvest vulnerability is positively correlated with elevation. Alternatively, snow may have increased elk detectability by hunters at higher elevation and thereby increased the likelihood of mortality at higher elevation. Clarify please.

*We have removed this sentence from the discussion as it does indeed contradict with the results. Survival was lower at higher elevation, as discussed in the remainder of the paragraph, and so this sentence was eliminated to maintain continuity with the results and the remainder of the discussion.*

Lines 383-392: Please reformulate this section in a more concise manner.

*We agree with the edits provided and appreciate the corrections that have improved clarity and made this section more concise. These lines have been adjusted as per the comments of the reviewer.*

Finally, I recommend you to acknowledge reviewers. I consider this good habit as one of the most important point of academic ethics

*We added a sentence to the acknowledgements thanking the editors and reviewers for their comments to our manuscript. Thank you for the time and consideration of our study, we believe the additional comments have greatly strengthened the manuscript.*
